# Non-enhanced CT-based radiomics signature of epicardial adipose tissue for screening coronary heart disease

**DOI:** 10.3389/fcvm.2026.1676562

**Published:** 2026-03-09

**Authors:** Yisen Deng, Zhan Liu, Xuming Wang, Xixi Gao, Zhaohua Zhang, Dingkai Zhang, Mingyuan Xu, Jianyan Wen, Peng Liu

**Affiliations:** 1 Department of Cardiovascular Surgery, China-Japan Friendship Hospital, Beijing, China; 2 Department of Lung Surgical Ward II, Shandong Cancer Hospital and Institute, Shandong First Medical University and Shandong Academy of Medical Sciences, Jinan, Shandong, China; 3 Department of Vascular Surgery, Shandong Provincial Hospital Affiliated to Shandong First Medical University, Jinan, Shandong, China

**Keywords:** coronary heart disease, epicardial adipose tissue, non-enhanced CT, radiomics, machine learning

## Abstract

**Objective:**

Our study aimed to establish a predictive model based on non-enhanced CT imaging features of epicardial adipose tissue (EAT) to differentiate patients with coronary heart disease (CHD) from those without.

**Methods:**

In this radiomics study, we collected clinical and radiomic data from a total of 281 patients diagnosed with CHD at the China-Japan Friendship Hospital, along with 188 healthy individuals who underwent physical examinations at our hospital. The participants were allocated to either a training or validation group at random, following a 7:3 ratio. We performed multivariate logistic regression analysis to create a clinical model, using a significance threshold of *p* < 0.05. Additionally, we employed the Least Absolute Shrinkage and Selection Operator (LASSO) algorithm to highlight important radiomic features for constructing a radiomics model. Lastly, we integrated the clinical and radiomics models to establish a combined model. To assess the model's effectiveness, we used the area under the curve (AUC), DeLong's test, and decision curve analysis (DCA).

**Results:**

In this radiomics study, the AUC of the clinical model were 0.883 (95% CI: 0.848–0.918) for the training cohort and 0.872 (95% CI: 0.812–0.932) for the validation cohort. In the radiomics model, the AUC for the training cohort was 0.853 (95% CI: 0.814–0.892) and for the validation cohort, it was 0.822 (95% CI: 0.751–0.893). DeLong's test revealed no significant difference in AUC between the clinical and radiomics models in both the training cohort (*p* = 0.218) and the validation cohort (*p* = 0.24). The combined model exhibited good discriminative ability, and the AUC were 0.930 (95% CI: 0.905–0.956) for the training cohort and 0.914 (95% CI: 0.863 −0.965) for the validation cohort. In the DeLong's test, we found that the AUC of the combined model was significantly higher in both cohorts compared to the other models (*p* < 0.05). Furthermore, the DCA curve revealed that using the combined model to identify patients with CHD provided greater advantages compared to using the two separate models.

**Conclusions:**

Our findings indicate that the combined model, which incorporated clinical features and the radiomics signature of EAT, can serve as a valuable tool for distinguishing patients with and without CHD.

## 1 Introduction

Over the past three decades, there has been a significant increase in the incidence of global cardiovascular disease (CVD) by 93%. A corresponding rise in mortality rates was also seen, accounting for approximately one-third of annual global deaths which surpasses the number of cancer-related deaths by more than twofold (1, 2). Among the various cardiovascular conditions, coronary artery atherosclerotic heart disease, commonly referred to as coronary heart disease (CHD), represents a crucial component. CHD is characterized by the narrowing or blockage of the coronary artery lumen due to inflammation, lipid deposition, or calcification, leading to the formation of atherosclerotic plaques. Consequently, this results in myocardial tissue ischemia, hypoxia, or necrosis, ultimately leading to coronary heart disease (3). CHD has emerged as one of the primary causes of death worldwide, despite numerous preventive measures implemented in recent years (4). Accumulating evidence has revealed a close association between ectopic fat accumulation, visceral fat, and the pathogenesis of CHD. Epicardial adipose tissue (EAT) has drawn considerable interest due to its crucial role in the onset, progression, and prognosis of coronary heart disease (CHD) (5, 6). EAT is located in direct proximity to the myocardium and nourished by minor branches of the coronary artery. It primarily comprises adipocytes, stromal vascular cells, fibroblasts, nerves, and different types of immune cells (7, 8). Unlike other adipose tissues, EAT lacks fascia separating it from adjacent myocardial or coronary artery walls. Consequently, secretory products from EAT can enter the coronary circulation through paracrine or vascular secretion mechanisms, influencing the function of coronary artery or myocardial cells (7, 9). EAT serves as a local energy reservoir for the heart, exhibiting high lipolytic and lipogenic activities. This enables cardiomyocytes to prevent excessive influx of free fatty acids (FFAs) and related lipotoxicity through fat generation. However, when the concentration of FFAs in the bloodstream surpasses the compensatory capacity of EAT, lipotoxicity can occur, leading to mitochondrial dysfunction, impaired oxidative metabolism, and increase oxidative stress (10). In addition to its energy storage role, EAT also possesses endocrine and immune functions (8, 11). EAT is now recognized as a highly active endocrine organ that produces cytokines, adipokines, and chemokines. These compounds can have both protective and harmful effects, influenced by the local microenvironmental conditions (12). Adipocytes within EAT play various immunological roles, participating in pathological processes associated with chronic inflammatory diseases (13). Interactions between different innate and acquired immune cells, as well as subsequent immunomodulation and activation of cytokine and chemokine secretion, have been found to contribute to the progression of atherosclerosis in experimental models (14). Recently, EAT has been considered a potential biomarker for atrial fibrillation (AF), acute coronary syndrome, and major adverse cardiac events (MACE) (15–17). Due to its unique location and multifaceted metabolic properties, EAT has become a new area of interest in research on cardiometabolic risk factors, exhibiting both systemic and local effects.

In recent years, radiomics has garnered increasing attention, which involves utilizing advanced image analysis techniques to extract extensive high-dimensional data from specific regions of interest (ROI) in digital images. These data are then converted into mineable information, allowing for the identification of phenotypically relevant features and the development of models to support physicians in improving diagnostic, predictive, and prognostic accuracy (18–21). Within the cardiovascular domain, radiomics has demonstrated its unique value in identifying coronary plaques and distinguishing between hypertensive heart disease and hypertrophic cardiomyopathy (22, 23). Notably, distinct imaging phenotypes of EAT have been observed in patients with and without CHD (24, 25). Verma et al. have suggested that EAT could serve as an attractive target for future interventions aimed at reducing cardiovascular risk (26). Furthermore, radiomics features of pericoronary adipose tissue (PCAT) derived from coronary artery computer tomography angiography (CCTA) have shown promise in predicting the occurrence of subsequent acute coronary syndromes. In fact, the comprehensive PCAT score outperforms the plaque score in identifying acute coronary syndromes within a three-year timeframe (27). This non-invasive radiomics model could help clinicians identify patients susceptible to MACE within three years, enabling them to implement preventive measures and intensive treatments before MACE occurs.

Most of the aforementioned studies have been conducted using CCTA; however, its application for mass population screening is limited due to concerns related to iodine contrast agents, testing costs, and other factors. Furthermore, iodized contrast media have been found to accelerate the decay of pericoronary fat in inflammatory conditions, which can affect its predictive capability. In contrast, non-enhanced computed tomography (CT) images may more reliably depict the true radiomics features (28). Additionally, some patients undergo coronary angiography for the diagnosis of CHD rather than CCTA. With the increasing emphasis on health, plain lung CT scans are becoming more prevalent in clinical physical examinations and various assessments. By leveraging the platform of CT plain scans, it may be possible to establish a radiomics model to discriminate patients with CHD from those without. This model could guide suspicious patients towards further examinations and treatment, effectively optimizing the utilization of clinical resources and reducing medical costs.

In this study, three predictive models were established and evaluated for their diagnostic efficacy. The aim was to create a prediction model utilizing the radiomics features of EAT in CT scans that could effectively differentiate patients with CHD from those without, identify high-risk groups, and ultimately enhance patient prognosis through initiating early preventive measures and timely interventions.

## 2 Method

### 2.1 Study population

This study included a total of 469 participants, comprising 281 patients diagnosed with CHD and 188 healthy individuals who underwent physical examinations at the China-Japan Friendship Hospital. CHD was defined as the presence of at least one major epicardial coronary artery with ≥50% luminal stenosis. Exclusion criteria were applied, including age over 80 years, history of thoracotomy surgery, severe infection, severe organ dysfunction, autoimmune diseases, malignant tumors, and poor CT image quality. The participants were randomly assigned to a training cohort of 337 individuals and a validation cohort of 132 individuals, following a 7:3 ratio. This study followed the principles set forth in the Declaration of Helsinki and obtained approval from the Medical Ethics Committee of China-Japan Friendship Hospital.

### 2.2 Population characteristics and CT scanning

Demographic characteristics and relevant examination data were retrieved from the hospital information system. CT examinations were conducted for all patients within one week before surgery. A multi-slice spiral CT system was utilized for the scanning with the following parameters: tube voltage of 120 kV, current of 300 mAs, and slice thickness of 5 mm. The scan covered the area from lung apex to the inferior border of the second lumbar spine.

### 2.3 EAT segmentation

Region delineation in this study was conducted with open-source 3D Slicer software (version 4.13.0). We segmented the total epicardial adipose tissue (EAT) volume, defined as the fat contained within the pericardial sac (Supplementary Figure 1). We did not specifically segment pericoronary adipose tissue (PCAT). Two cardiac surgeons independently delineated volumes of interest (VOIs) without access to clinical information. After completing the segmentation, we calculated the EAT volume and radiodensity wtih the software. One month later, another cardiac surgeon repeated the VOI delineation and then computed the average volume and radiodensity.

### 2.4 Feature extraction and selection

The voxel intensity values of the Volume of Interest (VOI) were normalized using *Z*-score standardization to reduce scanner-specific variations. All images were resampled to an isotropic voxel size of 1.0 × 1.0 × 1.0 mm^3^ using linear interpolation to ensure uniform feature calculation. Wavelet transforms (including eight combinations like HHL, LLL, etc.) and Laplacian of Gaussian (LoG) filters with sigma values of 1.0, 2.0, 3.0, 4.0, and 5.0 were applied to the original images to extract features at different spatial frequencies and scales. A total of 1218 quantitative features were initially extracted, encompassing shape, first-order statistics, and texture features [from Gray Level Co-occurrence Matrix (GLCM), Gray Level Run Length Matrix (GLRLM), etc.]. Radiomic features were extracted from the processed images using the PyRadiomicslibrary (version 3.0.1) in Python.

We normalized the radiomics features to address scale differences in both cohorts. We calculated both the intra-class and inter-class correlation coefficients (ICC) to assess the repeatability. Features with an ICC greater than 0.9 were deemed to exhibit strong repeatability and were included in subsequent analyses. After standardizing the data, we conducted a student's *t*-test to exclude radiomics features without statistical significance. Additionally, we performed redundancy analysis by calculating Pearson or Spearman correlation coefficients. Features with correlation coefficients greater than 0.9 were eliminated. The Least Absolute Shrinkage and Selection Operator (LASSO) algorithm with 5-fold cross-validation was employed for feature selection. The optimal penalty parameter (λ) was determined by the minimum binomial deviance criterion.

### 2.5 Construction of prediction models

Multivariate logistic regression analysis was used to evaluate the features of EAT, including EAT volume and radioactivity density. Features with *p* < 0.05 in the multivariate logistic regression analysis were utilized to establish the clinical model. Features with a *p*-value < 0.05 were used to establish the clinical model. The chosen radiomics features were weighted according to their coefficients and utilized to build a radiomics signature using linear modeling. We then developed a combined model by integrating features from both the radiomic and clinical models and constructed a nomogram to visually represent it.

### 2.6 Models performance evaluation

The diagnostic performance of the models was evaluated using the area under the receiver operating characteristic (ROC) curve (AUC), and DeLong's test was used to compare the AUCs of the models. We then used calibration curves and performed 1,000 bootstrap resampling validations to assess the alignment of the curve with the ideal standard. In addition, we conducted decision curve analysis (DCA) to quantify the net benefits at various threshold probabilities and to assess the clinical utility of the prediction model. Finally, we validated the three models in the validation cohort.

### 2.7 Statistical analysis

Statistical analyses were performed through R software (version 3.5.1) and SPSS (version 26.0). Continuous variables were reported as mean ± standard deviation (sd), while categorical variables were expressed as counts (percentages). *T*-tests were used to evaluate continuous variables, while chi-square tests were applied to categorical variables, to assess differences in clinical and CT imaging features between the training and validation cohorts. A two-sided *p*-value < 0.05 was regarded as statistically significant.

## 3 Result

### 3.1 Clinical features

**Figure 1:**
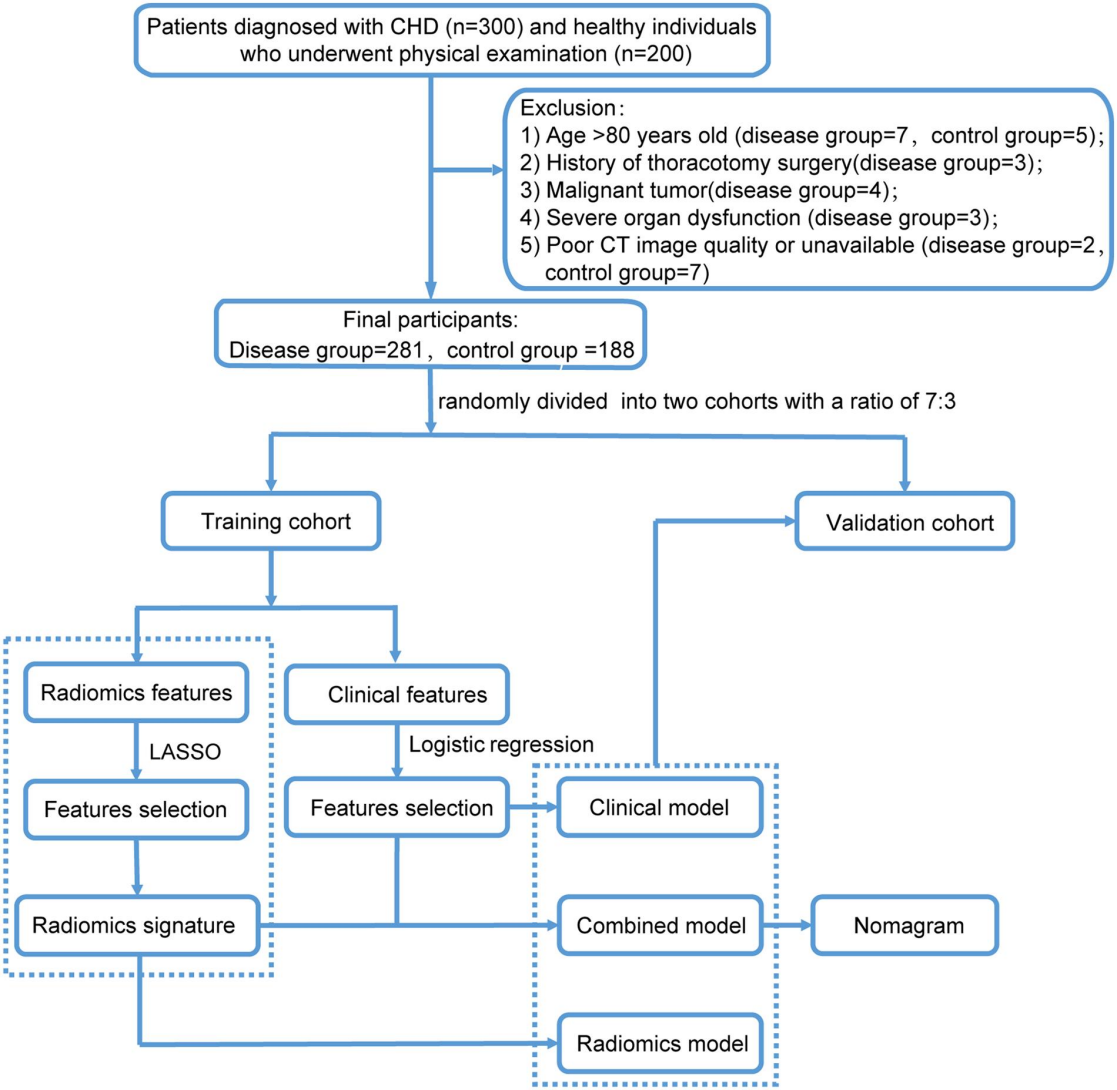
Flow chart illustrating the study process.

This study enrolled 469 participants, with 281 in the disease group and 188 in the control group. The study population was allocated into a training cohort (disease group: 200, control group: 137) and a validation cohort (disease group: 81, control group: 51). The study process is depicted in Figure 1, and the characteristics of the two groups are shown in Table 1. No significant differences were observed in the clinical characteristics between the training and validation cohorts (*P* > 0.05) (Supplementary Table 1).

**Table 1:** Clinical characteristic of the participants in this study.

Variables	Total (*n* = 469)	Disease group (*n* = 188)	Control group (*n* = 281)	*p*
Sex, *n* (%)				<0.001
Female	156 (33.3)	97 (51.6)	59 (21)	
Male	313 (66.7)	91 (48.4)	222 (79)	
Age	60.8 ± 11.1	57.2 ± 11.1	63.2 ± 10.4	<0.001
BMI (kg/m^2^)	25.5 ± 3.5	25.5 ± 3.5	25.5 ± 3.6	0.853
Smoking history, *n* (%)				<0.001
No	289 (61.6)	164 (87.2)	125 (44.5)	
Yes	180 (38.4)	24 (12.8)	156 (55.5)	
Alcohol abuse, *n* (%)				<0.001
No	387 (82.5)	176 (93.6)	211 (75.1)	
Yes	82 (17.5)	12 (6.4)	70 (24.9)	
HBP, *n* (%)				<0.001
No	221 (47.1)	131 (69.7)	90 (32)	
Yes	248 (52.9)	57 (30.3)	191 (68)	
Diabetes, *n* (%)				<0.001
No	335 (71.4)	173 (92)	162 (57.7)	
Yes	134 (28.6)	15 (8)	119 (42.3)	
Hyperlipidemia, *n* (%)				<0.001
No	289 (61.6)	168 (89.4)	121 (43.1)	
Yes	180 (38.4)	20 (10.6)	160 (56.9)	
Cerebrovascular disease, *n* (%)				<0.001
No	402 (85.7)	181 (96.3)	221 (78.6)	
Yes	67 (14.3)	7 (3.7)	60 (21.4)	
COPD, *n* (%)				0.033
No	457 (97.4)	187 (99.5)	270 (96.1)	
Yes	12 (2.6)	1 (0.5)	11 (3.9)	
WBC (10^9^/L)	7.2 ± 2.6	5.7 ± 1.5	8.2 ± 2.7	<0.001
Neutrophile (10^9^/L)	4.8 ± 2.5	3.4 ± 1.2	5.8 ± 2.6	<0.001
Lymphocyte (10^9^/L)	1.8 ± 1.2	1.8 ± 0.5	1.8 ± 1.5	0.84
Hemoglobin (g/L)	135.0 ± 19.2	137.4 ± 15.2	133.4 ± 21.4	0.028
Platelet (10^9^/L)	213.0 (180.0, 253.0)	215.5 (182.8, 253.2)	209.0 (175.0, 252.0)	0.295
ALT (U/L)	21.0 (15.0, 32.0)	18.0 (13.0, 24.0)	24.0 (17.0, 37.0)	<0.001
AST (U/L)	21.0 (17.0, 32.0)	19.0 (16.0, 23.0)	24.0 (18.0, 42.0)	<0.001
ALB (g/L)	42.0 ± 3.8	42.9 ± 3.1	41.4 ± 4.1	<0.001
CR (*μ*mol/L)	86.6 ± 80.4	68.2 ± 14.9	98.9 ± 101.4	<0.001
TC (mmol/L)	4.4 ± 1.3	4.5 ± 0.7	4.3 ± 1.5	0.099
TG (mmol/L)	1.7 ± 1.4	1.6 ± 1.1	1.7 ± 1.6	0.219
LDL (mmol/L)	2.7 ± 0.8	2.8 ± 0.5	2.7 ± 0.9	0.126
APTT (s)	37.9 ± 12.5	36.0 ± 3.7	39.3 ± 15.7	0.005
D-D (μg/mL)	0.6 ± 0.8	0.4 ± 0.4	0.7 ± 1.0	0.002
CKMB (U/L)	17.5 ± 37.5	11.0 ± 7.6	21.9 ± 47.6	0.002
EAT volume (cm^3^)	137.0 ± 48.6	123.1 ± 45.6	146.3 ± 48.4	<0.001
EAT density (Hu)	−75.3 ± 4.7	−75.9 ± 3.9	−75.0 ± 5.2	0.03

BMI, body mass index; HBP, high blood pressure; COPD, chronic obstructive pulmonary disease; WBC, white blood cell; ALT, alanine transaminase; AST, aspartate aminotransferase; ALB, albumin; CR, creatinine; TC, total cholesterol; TG, triglyceride; LDL, low density lipoprotein; APTT, activated partial thromboplastin time; D-D, d-dimer; CKMB, creatine kinase MB isoenzyme; EAT, epicardial adipose tissue.

### 3.2 Feature selection and construction of radiomic signature

**Figure 2:**
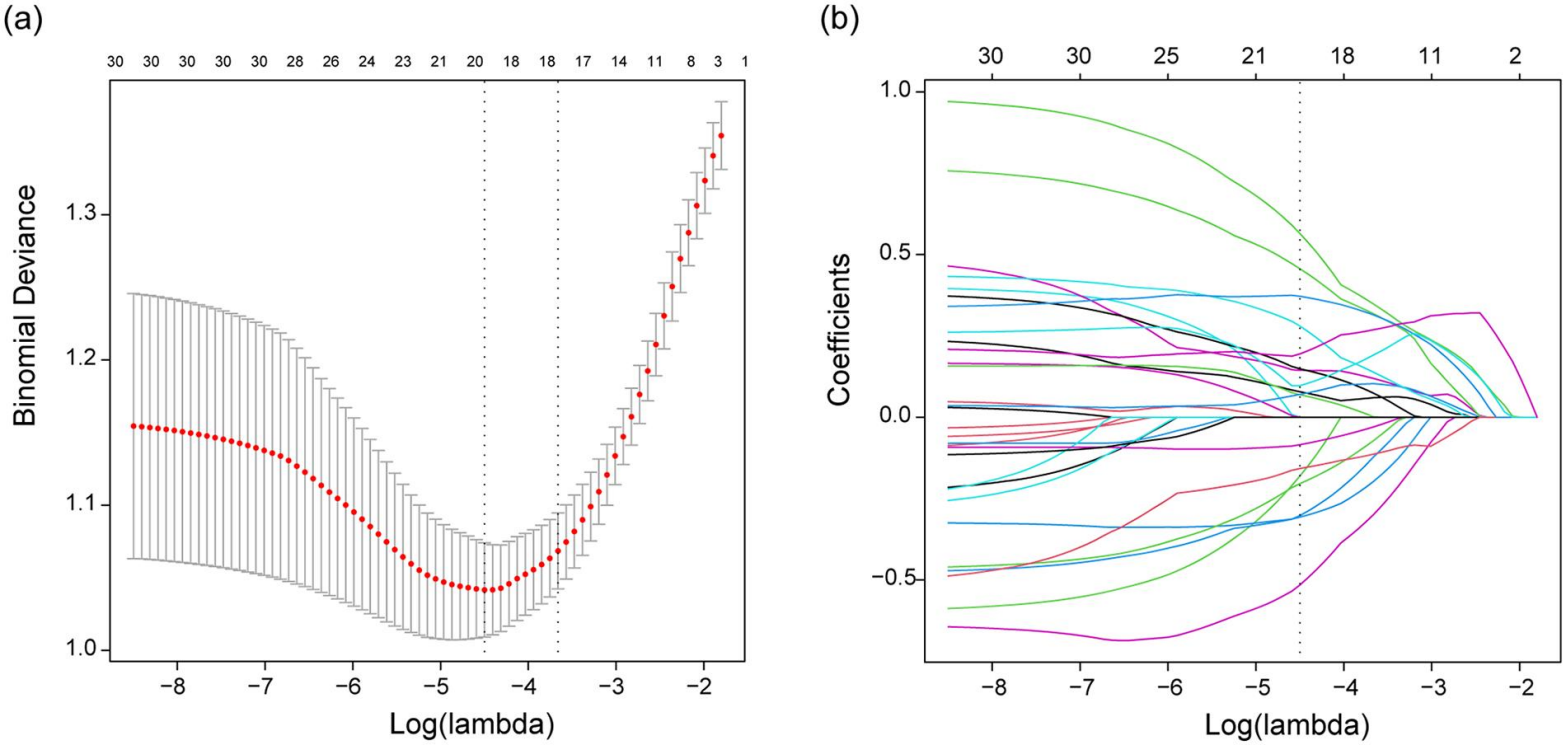
LASSO algorithm used for screening significant radiomics features. Mnimum criteria **(a)**, LASSO coefficient profiles **(b)**.

A total of 1,218 radiomic features were initially extracted from volume of interest (VOI), and 1,165 features with an intra-class correlation (ICC) value greater than 0.9 were retained for subsequent analysis. Redundant features with Spearman correlation coefficients exceeding 0.9 were subsequently excluded. Ultimately, the LASSO algorithm identified 18 significant radiomic features, which were used to construct the radiomic signature (Figures 2a,b). The calculation of the radiomic signature is as follows:

Radiomic signature=−0.54323703 -

original_shape_Maximum2DDiameterRow * 0.18086990 -

wavelet.HHL_glszm_ZoneEntropy * 0.30271098 +

wavelet.HLH_glcm_MaximumProbability * 0.14392601 +

wavelet.LLL_glcm_Idn * 0.45641397 +

wavelet.LLL_glszm_GrayLevelNonUniformity * 0.07816438 +

wavelet.HLL_glszm_ZoneEntropy * 0.06932275 -

wavelet.HHL_firstorder_Skewness * 0.30742317 -

log.sigma.2.0.mm.3D_glszm_SmallAreaLowGrayLevel@ Emphasis * 0.08466977 +

wavelet.HHL_firstorder_Median * 0.14863790 -

wavelet.LHH_firstorder_Skewness * 0.20457687 +

original_glszm_ZoneEntropy * 0.07013460 +

original_shape_LeastAxisLength * 0.28005880 -

wavelet.HLH_glcm_Imc1 * 0.51574311 -

wavelet.HHL_firstorder_RootMeanSquared * 0.15779457 +

original_shape_MajorAxisLength * 0.56483267+

wavelet.LLL_firstorder_RootMeanSquared * 0.37179138 +

wavelet.HHL_glszm_GrayLevelNonUniformityNormalized * 0.09678572+log.sigma.1.0.mm.3D_glcm_Idn * 0.19443036.

### 3.3 Development and validation of predictive models

**Figure 3:**
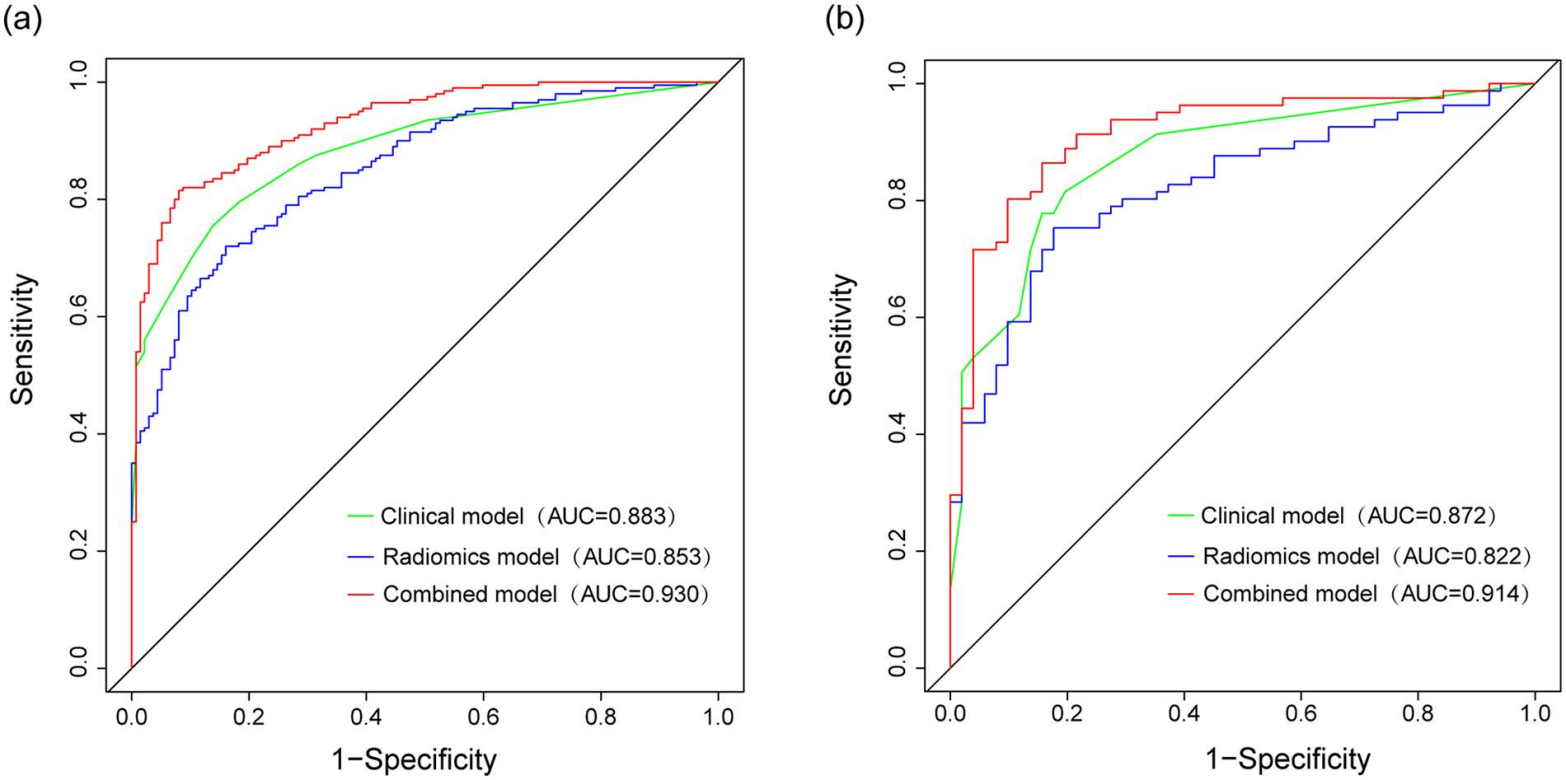
Receiver operating characteristic (ROC) curves comparing the performance of the clinical, radiomics, and combined models in the training **(a)** and validation **(b)** cohorts.

Multivariate logistic regression analysis revealed smoking, hypertension, diabetes, and hyperlipidemia as independent risk factors for CHD in the training cohort (Table 2). The clinical model included only these independent risk factors with significant differences in the multivariate analysis. As the CHD patients treated in hospital usually presented abnormal laboratory indicators, and we aimed to establish a combined model for discriminating patients with CHD from those without, laboratory indicators were not included in the clinical model. Therefore, the clinical model was developed using four independent risk factors: smoking, hypertension, diabetes, and hyperlipidemia. The AUCs of the clinical model were 0.883 (95%CI: 0.848–0.918) and 0.872 (95%CI: 0.812–0.932) in the training and validation cohorts, respectively (Figures 3a,b). The radiomics model exhibited AUCs of 0.853 (95%CI: 0.814–0.892) and 0.822 (95%CI: 0.751–0.893) in the training and validation cohorts, respectively (Figures 3a,b). DeLong's test indicated that the AUCs of the clinical model and radiomics model did not differ significantly in the training cohort (*p* = 0.218) and validation cohort (*p* = 0.24).

**Table 2:** Multivariate logistic regression analysis of the training cohort.

Variables	n. total	n. event（%）	OR (95%CI)	*P* value
Sex				
Female	113	45 (39.8)	1 (Ref)	
Male	224	155 (69.2)	0.64 (0.07∼5.83)	0.695
Age	337	200 (59.3)	1.06 (0.99∼1.12)	0.078
Smoking history				
No	210	93 (44.3)	1(Ref)	
Yes	127	107 (84.3)	16.34 (2.5∼106.82)	0.004
Alcohol abuse				
No	282	155 (55)	1 (Ref)	
Yes	55	45 (81.8)	0.76 (0.07∼8.38)	0.822
HBP				
No	160	66 (41.2)	1 (Ref)	
Yes	177	134 (75.7)	5.49 (1.42∼21.21)	0.013
Diabetes				
No	237	112 (47.3)	1 (Ref)	
Yes	100	88 (88)	19.76 (3.07∼127.38)	0.002
Hyperlipidemia				
No	204	81 (39.7)	1 (Ref)	
Yes	133	119 (89.5)	17.75 (3.58∼87.92)	<0.001
Cerebrovascular disease				
No	294	164 (55.8)	1 (Ref)	
Yes	43	36 (83.7)	2.04 (0.17∼25.04)	0.577
COPD				
No	327	191 (58.4)	1 (Ref)	
Yes	10	9 (90)	7.9 (0.01∼9135.16)	0.566
EAT volume	337	200 (59.3)	1 (0.98∼1.01)	0.569
EAT density	337	200 (59.3)	1.12 (0.93∼1.34)	0.226

HBP, high blood pressure; COPD, chronic obstructive pulmonary disease; EAT, epicardial adipose tissue.

**Figure 4:**
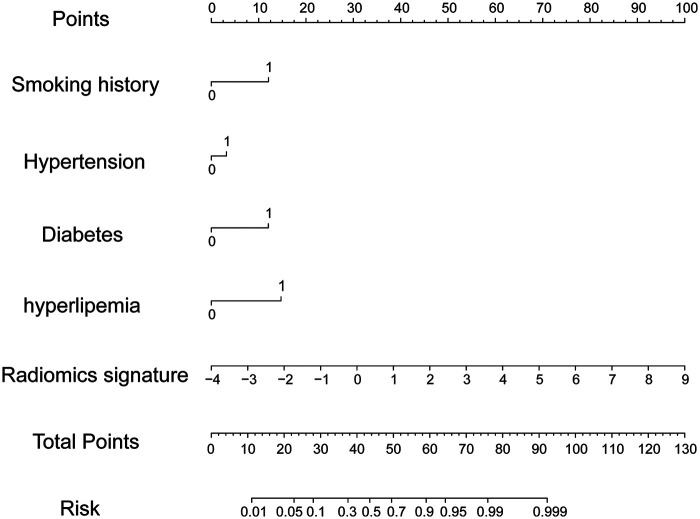
Nomogram representing the combined model. Note: Locate the patient's value for each radiomic feature, draw a line upward to the “Points” axis to determine the score for each variable, sum all the points, and then draw a line down from the “Total Points” axis to the “Risk of CHD” axis to obtain the individual probability of coronary heart disease).

The combined model, which incorporated both clinical features and the radiomics signature, was represented in a nomogram (Figure 4). The model demonstrated strong discriminant ability, with AUCs of 0.930 (95%CI: 0.905–0.956) in the training cohort and 0.914 (95%CI: 0.863–0.965) in the validation cohort (Figures 3a,b). Sensitivity, specificity, accuracy, PPV (positive predictive value), and NPV (negative predictive value) with confidence intervals of the training cohort and validation cohort were calculated (Supplementary Tables 2, 3**)**.

**Figure 5:**
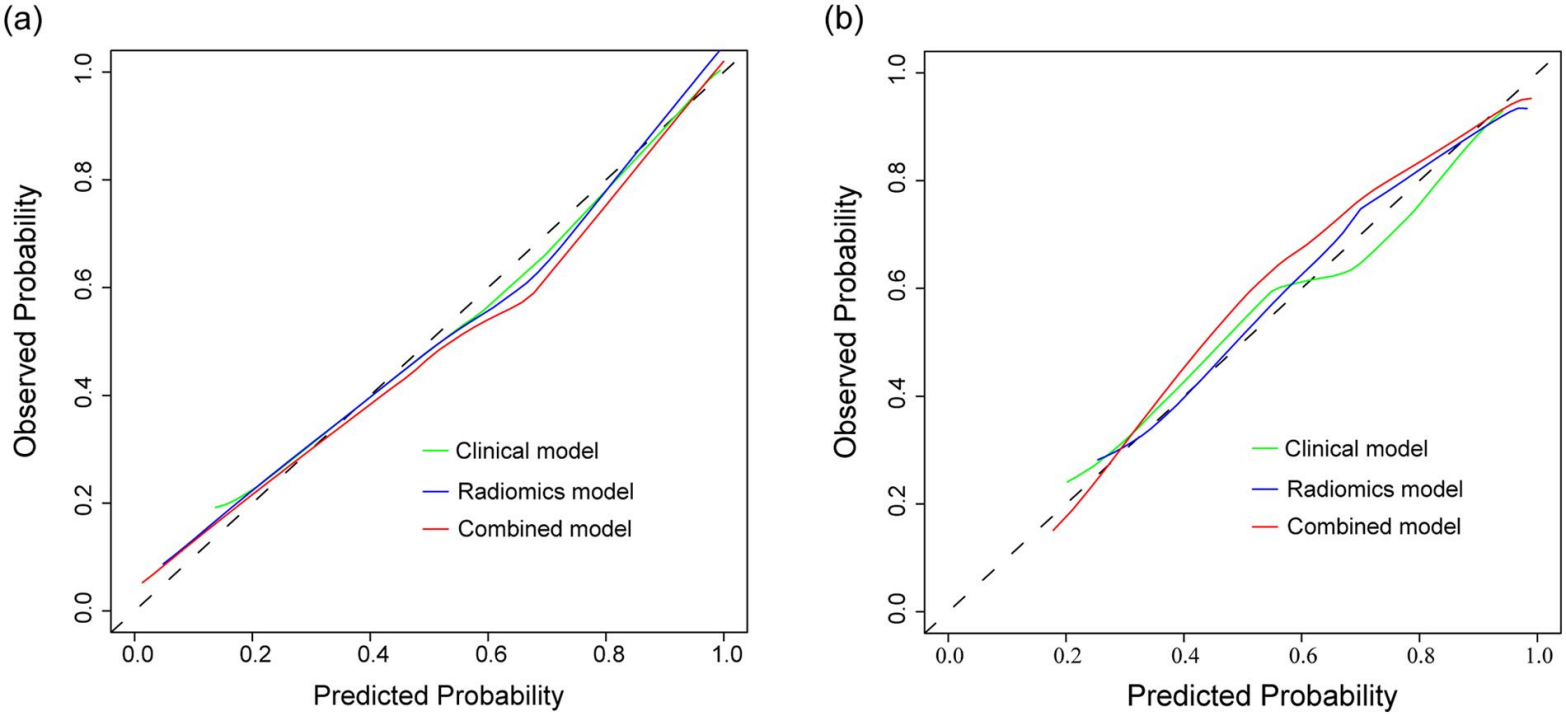
Calibration curves demonstrating the agreement between predicted and observed probabilities of the clinical, radiomics, and combined models in the training **(a)** and validation **(b)** cohorts.

**Figure 6:**
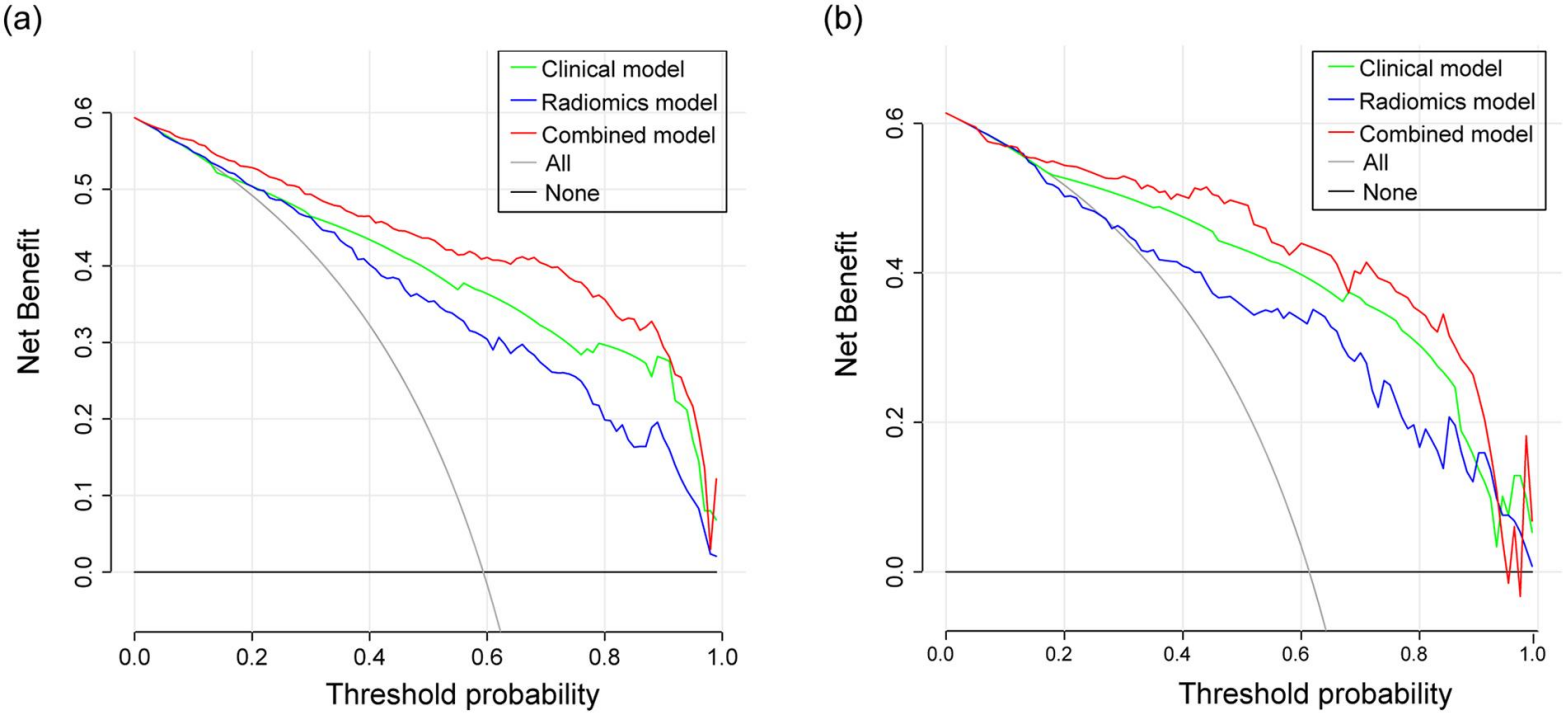
Decision curve analysis showing the clinical utility of the clinical, radiomics, and combined models in the training **(a)** and validation **(b)** cohorts.

DeLong's test showed that the combined model outperformed both the clinical model (*p* < 0.001) and radiomics model (*p* < 0.001) in the training cohort. In the validation cohort, similar results were observed, with the combined model showing superiority over the clinical model (*p* = 0.003) and the radiomics model (*p* = 0.001). The calibration curve demonstrated good agreement between the training and validation cohorts (Figures 5a,b). Additionally, the decision curve analysis (DCA) curve illustrated that using the combined model to identify CHD patients had greater clinical advantages than using the two separate models (Figures 6a,b).

## 4 Discussion

Radiomics uses advanced image analysis techniques to extract high-dimensional data from digital images, revealing relationships between various indicators and diseases, which can help guide clinical decision-making. Clinical features from individuals with CHD and healthy controls, along with EAT radiomics features from non-enhanced CT scans were gathered in the study. Subsequently, three predictive models for CHD were developed and assessed: the clinical model, the radiomics model, and the combined model. The radiomics model and the clinical model showed similar discriminative abilities. However, the combined model exhibited the strongest predictive ability among the three models and displayed promising potential for identifying CHD patients. The superior performance of the combined model over the clinical model suggests that the EAT radiomics signature captures pathophysiological information related to CHD that is not fully explained by traditional clinical risk factors alone. Features quantifying heterogeneity (like Entropy) may reflect variations in adipocyte size, increased vascularity, immune cell infiltration, or fibrotic changes within the EAT. This textural heterogeneity is a surrogate for tissue-level inflammation and remodeling, which are central to the pathogenesis of coronary atherosclerosis. In the future, there may be an opportunity to utilize the combined model to discriminate patients with CHD from those without.

The application of radiomics in the cardiovascular field continues to expand, leading to significant advancements. For instance, radiomics has facilitated the identification of functional coronary artery stenosis, the detection of the napkin ring sign (a low attenuation area of plaque surrounded by a ring of high attenuation area), and the analysis of correlations between the phenotype of PCAT and adverse events such as myocardial infarction (22, 29, 30). EAT exhibits a strong correlation with cardiovascular diseases, including arrhythmia, coronary artery disease, and heart failure (31–33). Data from large longitudinal population studies have demonstrated that EAT volume independently associates with the incidence of myocardial infarction (34). Beyond its volume, the density of EAT observed on CT scans also holds significant importance, as it indicates elevated vascularity and mitochondrial content within the fat tissue (35). Studies have confirmed that increased CT density based on PCAT is associated with heart-related mortality, increased plaque load, and an elevated risk of abnormal myocardial perfusion (36–39). The multivariate analysis indicated that EAT volume and density did not vary notably across the disease group and the control group in this study; therefore, they were not included in the prediction model. Eisenberg et al. reported that measurements of EAT volume and attenuation could help predict MACE in asymptomatic subjects, independently of traditional risk factors (40). While these studies have focused on EAT volume and attenuation, further research is required to explore the microscopic distribution of EAT and its application based on significant radiomics features.

Pericoronary fat, which serves as a source of inflammatory mediators in the coronary circulation, has been implicated in the development of coronary artery-related diseases (41–44). In addition to dyslipidemia, inflammation has been recognized as a significant contributor to the pathophysiology of atherosclerotic formation and ischemic events (2). Vascular inflammation is considered a key factor in the formation and instability of atherosclerotic plaques (45, 46). The bidirectional connection between the coronary artery wall and perivascular adipose tissue (PVAT) enables the secretion of proinflammatory cytokines within the vascular wall, inhibiting the differentiation and lipid accumulation of PVAT pre-adipocytes under inflammatory conditions. This creates a gradient in PVAT composition, ranging from a fatty and less watery phase near the non-focal artery to a lipid-deficient and more watery phase near the inflamed artery (47, 48). The changes in PVAT composition caused by inflammation result in an increase in CT attenuation from more negative values (near −190 HU) to less negative values (near −30 HU). This dynamic alteration in the balance of water and fat content is reflected by the fat attenuation index (FAI) (49), which serves as a sensitive and non-invasive biomarker of coronary inflammation closely associated with the occurrence of coronary events, plaque type, and patient prognosis (36, 50–52). Oikonomou et al. initially proposed that machine learning models based on radiomics features of pericoronary fat might significantly enhance the prediction of cardiac risk (53). They introduced a fat radiomic profile (FRP) based on coronary PVAT features derived from CCTA and assessed it across several study cohorts. Their findings suggest that FRP has the potential to improve the prediction of cardiac risk for MACE beyond traditional risk factors. The machine learning models they established based on radiomics were able to distinguish cases with 5-year MACE from non-MACE cases, thus significantly enhancing the prediction of MACE risk in patients with coronary heart disease (53).

Another study found that the CCTA-based PCAT radiomics score was superior to the plaque score in predicting acute coronary syndrome (ACS) within the following 3 years. The radiomics score achieved an AUC of 0.826 in the training cohort and 0.811 in the test cohort, while the AUCs for the plaque scores were 0.699 and 0.640, respectively (27). Most radiomics studies on cardiovascular diseases have focused on the correlation between CCTA and the prognosis of coronary artery disease. CCTA provides strong diagnostic performance for severe coronary artery blockages (>50%) and is highly effective in excluding significant coronary conditions (54, 55). However, since 20% of CHD patients lack traditional risk factors and 40% have only one risk factor, CCTA is primarily recommended for patients with relevant symptoms or significant risk factors. Consequently, patients without obvious risk factors or CHD-related symptoms may miss the opportunity for early prevention and treatment if coronary atherosclerotic lesions are not detected promptly (56, 57). Moreover, the use of iodized contrast media in CCTA can increase the attenuation of pericoronary fat during inflammatory states, potentially affecting its predictive ability, whereas non-enhanced CT scan images to some extent reflect true image features (28). Nonetheless, there are limited studies based on non-enhanced CT in the cardiovascular field. Coronary artery calcium (CAC) scores have been employed to stratify cardiovascular risk and measure the burden of coronary atherosclerosis. It has been shown that total CAC scores measured by non-enhanced CT can better predict cardiovascular events compared to standard cardiovascular risk factors (58–61). To better contextualize the diagnostic performance of our non-contrast CT-based EAT radiomics model, we benchmarked our results against recent studies in this emerging field. For example, Tong et al. developed an EAT radiomics model for diagnosing coronary slow flow, reporting AUCs of 0.81 and 0.77 in the training and validation cohorts, respectively (62). In a larger multi-center study, Yu et al. constructed a model to predict myocardial ischemia, which demonstrated an AUC of 0.838 in an external validation set (63). Furthermore, Cohen-Doret al. achieved impressive results for predicting atrial fibrillation using left atrial EAT radiomics, with AUCs up to 0.88 using a logistic regression model (64). While these studies focus on distinct cardiovascular outcomes, they collectively demonstrate the utility of EAT radiomics derived from non-contrast CT. The AUC of 0.914 achieved by our combined model in the validation cohort compares favorably with these benchmarks. This suggests that our model, which integrates clinical features with a radiomics signature from routinely acquired non-contrast chest CT, possesses robust discriminatory power for identifying CHD. The consistently strong performance of EAT radiomics across different cardiovascular conditions further highlights the biological plausibility of EAT as a rich source of imaging biomarkers. Therefore, establishing a prediction model using radiomics features on non-enhanced CT for distinguishing CHD status is a promising endeavor.

This study has several limitations. Firstly, as a retrospective analysis conducted at one center, it may be prone to selection bias, and high prevalence of CHD can lead to optimistic performance estimates compared to a real-world screening scenario. Thus, external validation in a prospective, consecutive cohort with a disease prevalence reflective of the target screening population is necessary. Secondly, variations in in scanning protocols and segmentation methods for EAT images may impact the research results. Establishing a widely recognized delineation method for the EAT region could reduce heterogeneity in future studies. Lastly, The univariate feature filtering based on statistical significance with outcome labels was performed prior to dataset splitting. While this was a pragmatic step for high-dimensionality reduction, it may introduce a degree of optimism in the subsequent validation performance. Although bootstrap validation was employed, the stability of the radiomic signature could be further confirmed in future studies using repeated cross-validation with multiple data splits, which would provide an even more robust assessment of model performance. Prospective multicenter studies that include comparisons with established risk scores and cost analyses are necessary to improve the model's applicability across diverse settings before its application in clinical practice.

## 5 Conclusion

We have successfully established and evaluated three predictive models for CHD using clinical features and radiomics-derived characteristics of EAT obtained from non-enhanced CT. The combined model demonstrated superior performance, highlighting its potential as a valuable tool for distinguishing CHD patients. In the future, the combined model holds promise for aiding in the discrimination of patients with and without CHD.

## Data Availability

The original contributions presented in the study are included in the article/Supplementary Material, further inquiries can be directed to the corresponding authors.

## Supplementary material

SUPPLEMENTARY FIGURE 1

EAT segmentation within the pericardial sac. ROI segmentation **(a)**; 3D visualization **(b)**.
