# Supplementary material for: Non-enhanced CT-based radiomics signature of epicardial adipose tissue for screening coronary heart disease
Source: Front Cardiovasc Med. 2026 Mar 9;13:1676562. doi: 10.3389/fcvm.2026.1676562 (PMC13006323; doi:10.3389/fcvm.2026.1676562)
Supplement: Supplementary file 1 [file Table1.pdf]

Supplemental table 1. Comparison of clinical characteristics between the training cohort and validation cohort.

Variables	Total (n = 469)	Training cohort (n = 132)	Validation cohort (n = 337)	*p*
Sex, n (%)				0.843
Female	156 (33.3)	43 (32.6)	113 (33.5)	
Male	313 (66.7)	89 (67.4)	224 (66.5)	
Age	60.8 ± 11.1	59.6 ± 11.3	61.2 ± 11.0	0.143
BMI（kg/m^2）	25.5 ± 3.5	25.5 ± 3.4	25.5 ± 3.6	0.947
Smoking history, n (%)				0.621
No	289 (61.6)	79 (59.8)	210 (62.3)	
Yes	180 (38.4)	53 (40.2)	127 (37.7)	
Alcohol abuse, n (%)				0.289
No	387 (82.5)	105 (79.5)	282 (83.7)	
Yes	82 (17.5)	27 (20.5)	55 (16.3)	
HBP, n (%)				0.805
No	221 (47.1)	61 (46.2)	160 (47.5)	
Yes	248 (52.9)	71 (53.8)	177 (52.5)	
Diabetes, n (%)				0.399
No	335 (71.4)	98 (74.2)	237 (70.3)	
Yes	134 (28.6)	34 (25.8)	100 (29.7)	
Hyperlipidemia, n (%)				0.289
No	387 (82.5)	105 (79.5)	282 (83.7)	
Yes	82 (17.5)	27 (20.5)	55 (16.3)	
Cerebrovascular disease, n (%)				0.131
No	402 (85.7)	108 (81.8)	294 (87.2)	
Yes	67 (14.3)	24 (18.2)	43 (12.8)	
COPD, n (%)				0.523
No	457 (97.4)	130 (98.5)	327 (97)	
Yes	12 (2.6)	2 (1.5)	10 (3)	
WBC, (10^9/L)	7.2 ± 2.6	7.0 ± 2.2	7.3 ± 2.8	0.448
Neutrophile (10^9/L)	4.8 ± 2.5	4.7 ± 2.1	4.9 ± 2.6	0.363
Lymphocyte(10^9/L)	1.8 ± 1.2	1.7 ± 0.5	1.8 ± 1.4	0.558

Hemoglobin(g/L)	135.0 ± 19.2	134.2 ± 18.2	135.3 ± 19.6	0.591
Platelet(10^9/L)	213.0 (180.0, 253.0)	218.5 (186.0, 264.0)	211.0 (174.0, 251.0)	0.085
ALT(U/L)	21.0 (15.0, 32.0)	20.0 (15.8, 30.2)	21.0 (15.0, 33.0)	0.395
AST(U/L)	21.0 (17.0, 32.0)	21.0 (17.0, 30.0)	21.0 (17.0, 32.0)	0.799
ALB(g/L)	42.0 ± 3.8	41.9 ± 3.2	42.0 ± 4.0	0.83
CR（μmol/L）	86.6 ± 80.4	86.9 ± 109.8	86.4 ± 65.6	0.954
TC（mmol/L）	4.4 ± 1.3	4.5 ± 1.6	4.3 ± 1.1	0.113
TG（mmol/L）	1.7 ± 1.4	1.8 ± 1.8	1.6 ± 1.2	0.444
LDL（mmol/L）	2.7 ± 0.8	2.8 ± 0.9	2.7 ± 0.8	0.48
APTT（s）	37.9 ± 12.5	37.2 ± 6.0	38.2 ± 14.3	0.456
D-D（μg/ml）	0.6 ± 0.8	0.6 ± 0.8	0.6 ± 0.8	0.839
CKMB（U/L）	17.5 ± 37.5	17.5 ± 41.4	17.5 ± 36.0	0.994
EAT volume	137.0 ± 48.6	134.8 ± 47.0	137.8 ± 49.2	0.538
EAT density	-75.3 ± 4.7	-75.0 ± 4.4	-75.5 ± 4.8	0.359

Abbreviation: BMI, body mass index; HBP, high blood pressure; COPD, chronic obstructive pulmonary disease; WBC, white blood cell; ALT, alanine transaminase; AST, aspartate aminotransferase; ALB, albumin; CR, creatinine; TC, total cholesterol; TG, triglyceride; LDL, low density lipoprotein; APTT, activated partial thromboplastin time; D-D, d-dimer; CKMB, creatine kinase MB isoenzyme; EAT, epicardial adipose tissue.
