# Supplementary material for: Non-enhanced CT-based radiomics signature of epicardial adipose tissue for screening coronary heart disease
Source: Front Cardiovasc Med. 2026 Mar 9;13:1676562. doi: 10.3389/fcvm.2026.1676562 (PMC13006323; doi:10.3389/fcvm.2026.1676562)
Supplement: Supplementary file 2 [file Table2.pdf]

Table 2. Sensitivity, specificity, accuracy, PPV and NPV with confidence intervals of the training cohort.

	Auc	sensitivity	specificity	ACC	PPV	NPV
Clinical model	0.883	0.755(0.695-0.815)	0.861(0.803-0.919)	0.798(0.797-0.799)	0.888(0.841-0.936)	0.707(0.638-0.776)
Radiomics model	0.853	0.720(0.658-0.782)	0.839(0.778-0.901)	0.769(0.768-0.770)	0.867(0.816-0.919)	0.673(0.602-0.743)
Combined model	0.93	0.815(0.761-0.869)	0.920(0.874-0.965)	0.858(0.857-0.858)	0.937(0.901-0.973)	0.773(0.709-0.837)

Abbreviation: ACC, accuracy; PPV, positive predictive value; NPV, negative predictive value.
