# Supplementary material for: Non-enhanced CT-based radiomics signature of epicardial adipose tissue for screening coronary heart disease
Source: Front Cardiovasc Med. 2026 Mar 9;13:1676562. doi: 10.3389/fcvm.2026.1676562 (PMC13006323; doi:10.3389/fcvm.2026.1676562)
Supplement: Supplementary file 3 [file Table3.pdf]

Table 3. Sensitivity, specificity, accuracy, PPV and NPV with confidence intervals of the validation cohort.

	Auc	sensitivity	specificity	ACC	PPV	NPV
Clinical model	0.872	0.778(0.687-0.868)	0.843(0.743-0.943)	0.803(0.801-0.805)	0.887(0.814-0.961)	0.705(0.590-0.819)
Radiomics model	0.822	0.753(0.659-0.847)	0.824(0.719-0.928)	0.780(0.778-0.783)	0.871(0.793-0.950)	0.677(0.561-0.794)
Combined model	0.914	0.864(0.790-0.939)	0.843(0.743-0.943)	0.856(0.854-0.858)	0.897(0.830-0.965)	0.796(0.689-0.904)

Abbreviation: ACC, accuracy; PPV, positive predictive value; NPV, negative predictive value.
