# Supplementary figures and images for: Non-enhanced CT-based radiomics signature of epicardial adipose tissue for screening coronary heart disease
Source: Front Cardiovasc Med. 2026 Mar 9;13:1676562. doi: 10.3389/fcvm.2026.1676562 (PMC13006323; doi:10.3389/fcvm.2026.1676562)

(a)

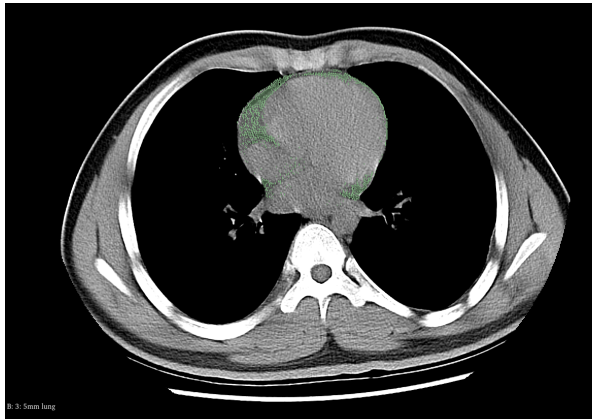

(b)

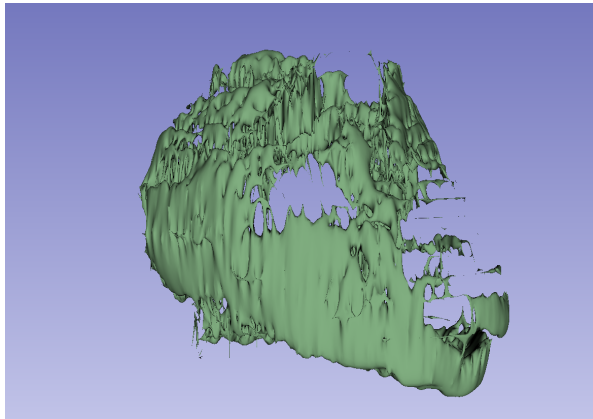

Supplement: Supplementary file 4 [file Image1.pdf]
